# The superior prognostic role of pRB1 over p16 immunohistochemistry as a biomarker in meningiomas

**DOI:** 10.1186/s40478-026-02419-3

**Published:** 2026-09-03

**Authors:** Hanna Gött, Jonas Tellermann, Hannes Becker, Christina Fodi, Peter Paßlack, Elgin Hoffmann, Maria Elisa Di Francesco, Daniel J. Merk, Jürgen Honegger, Ghazaleh Tabatabai, Marcos Tatagiba, Felix Behling, Jens Schittenhelm

**Affiliations:** 1 Center for Neuro-Oncology, Comprehensive Cancer Center Tübingen-Stuttgart, University Hospital Tübingen, Eberhard-Karls-University Tübingen, Tübingen, Germany; 2 Department of Neurosurgery, University Hospital Tübingen, Eberhard-Karls-University Tübingen, Tübingen, Germany; 3 Department of Neurology and Interdisciplinary Neuro-Oncology, University Hospital Tübingen, Eberhard-Karls-University Tübingen, Tübingen, Germany; 4 Cluster of Excellence (EXC 2180) “Image Guided and Functionally Instructed Tumor Therapies”, Eberhard-Karls University Tübingen, Tübingen, Germany; 5 Department of Anesthesiology, University Hospital Tübingen, Eberhard-Karls-University Tübingen, Tübingen, Germany; 6 Department of Radiation Oncology, University Hospital Tübingen, Eberhard-Karls-University Tübingen, Tübingen, Germany; 7 Department of Ophthalmology, Inselspital Bern, University of Bern, Bern, Switzerland; 8 German Cancer Consortium (DKTK), DKFZ Partner Site Tübingen, Tübingen, Germany; 9 Department of Neurosurgery, Faculty of Medicine, Augsburg University, Augsburg, Germany; 10 Translational Oncological Neurosurgery, Faculty of Medicine, University Augsburg, Augsburg, Germany; 11 Department of Neuropathology, Institute of Pathology and Neuropathology, University Hospital Tübingen, Eberhard-Karls-University Tübingen, Calwerstr. 3, 72076 Tübingen, Germany

**Keywords:** Meningioma, Tissue microarray, Immunohistochemistry, p16, pRB1, Prognosis, Recurrence-free survival, Progression-free survival

## Abstract

**Background:**

Despite best clinical management, patients with meningiomas frequently experience tumor recurrence. During recent decades, efforts have been made to improve the prognostic stratification of meningiomas by incorporating molecular data. A subgroup of tumors harboring a homozygous *CDKN2A* deletion was identified, and a higher risk of tumor progression was observed, suggesting the potential use of cyclin-dependent kinases as biomarkers.

**Material and methods:**

In this retrospective single-center study, the immunohistochemical staining for the cyclin-dependent kinases p16, phosphoRB1 (pRB1), CDK4 and CDK6 was analyzed in 1751 paraffin-embedded meningioma samples. For the assessment of p16, CDK4 and CDK6, a semi-quantitative score was applied, whereas an automated quantification tool was used for pRB1. The distribution and association with histopathological results, clinical data and progression-free survival (PFS)—defined by radiographic tumor recurrence—were assessed.

**Results:**

Of all meningioma samples, 14.9% (*n* = 261) expressed p16. Elevated pRB1 levels were found in 34.5% (*n* = 589) of tumor samples. CDK4 and CDK6 positive staining was observed in 41.9% and 42.2% of cases, respectively. Dichotomous stratification of meningiomas based on p16 and pRB1 expression levels suggested a significant influence on PFS in univariate analyses. Multivariate analysis determined elevated pRB1 expression, WHO grading, extent of resection, adjuvant radiotherapy, male gender, NF2-status and an elevated MIB1 index as independent prognostic factors.

**Conclusions:**

High expression of pRB1 is an independent prognostic factor for shorter PFS. The prognostic impact of p16 is mostly attributed to the confounding increase of pRB1 expression.

**Supplementary Information:**

The online version contains supplementary material available at https://doi.org/10.1186/s40478-026-02419-3.

## Introduction

Meningiomas are the most common primary intracranial tumors originating from prostaglandin D synthase-positive meningeal precursor cells of the meninges. The majority of meningiomas are benign and amenable to surgical resection alone. However, approximately 20% of meningiomas show more aggressive or even malignant characteristics, resulting in a higher rate of tumor recurrence and impaired overall survival [1–4]. Based on the histopathological grading and molecular data, the fifth edition of the WHO Classification of Tumors of the Central Nervous System differentiates 15 meningioma variants. Most of them are classified as CNS WHO grade 1 in absence of histological atypia, while fewer histological variants are allotted the CNS WHO grade 2 based on their morphology and known increased risk of recurrence. CNS WHO grade 3 meningiomas are either classified by their mitotic activity, presence of cell anaplasia or when specific molecular drivers (such as TERT promotor mutations or homozygous *CDKN2A/B* deletions) are present [5, 6]. Currently, surgical excision and radiotherapy are the only established treatment options for meningioma patients [7]. Therefore, the identification of cases with a higher risk of tumor recurrence is crucial for adjusting the follow-up management accordingly and considering early postoperative radiation therapy. The progression free survival is influenced by the extent of tumor resection and several other established factors such as brain invasion, *NF2* tumor mutation status, Chromosomal 1p deletion, *CDKN2A/B* homozygous deletion, *TERT* promoter mutation, *BAP1* alterations (associated with rhabdoid/papillary histology) and loss of H3K27me3 expression [5, 8–15]. Despite these advances, not all high-risk meningiomas are reliably stratified using these biomarkers and extensive molecular profiling is not available for the majority of samples operated worldwide. Recently, several integrative molecular classification systems for meningiomas were introduced, defining prognostically distinct molecular subgroups associated with different mutational, cytogenetic, and gene expression patterns. Consequently, these classifiers allow for a risk-adapted radiotherapy stratification of patients [16]. Data from such studies show that meningiomas with highly perturbed genomes, CNS WHO grade 3 tumors or with a methylation signature of malignant meningiomas are associated with CDKN2A homozygous deletion in up to 27%, while *CDKN2A* deletion is present only in 3% of tumors with histological signs of atypia according to CNS WHO grade 2 and is almost exclusively with histological grading of CNS WHO Grade 1 [5, 13].

**Fig. 1:**
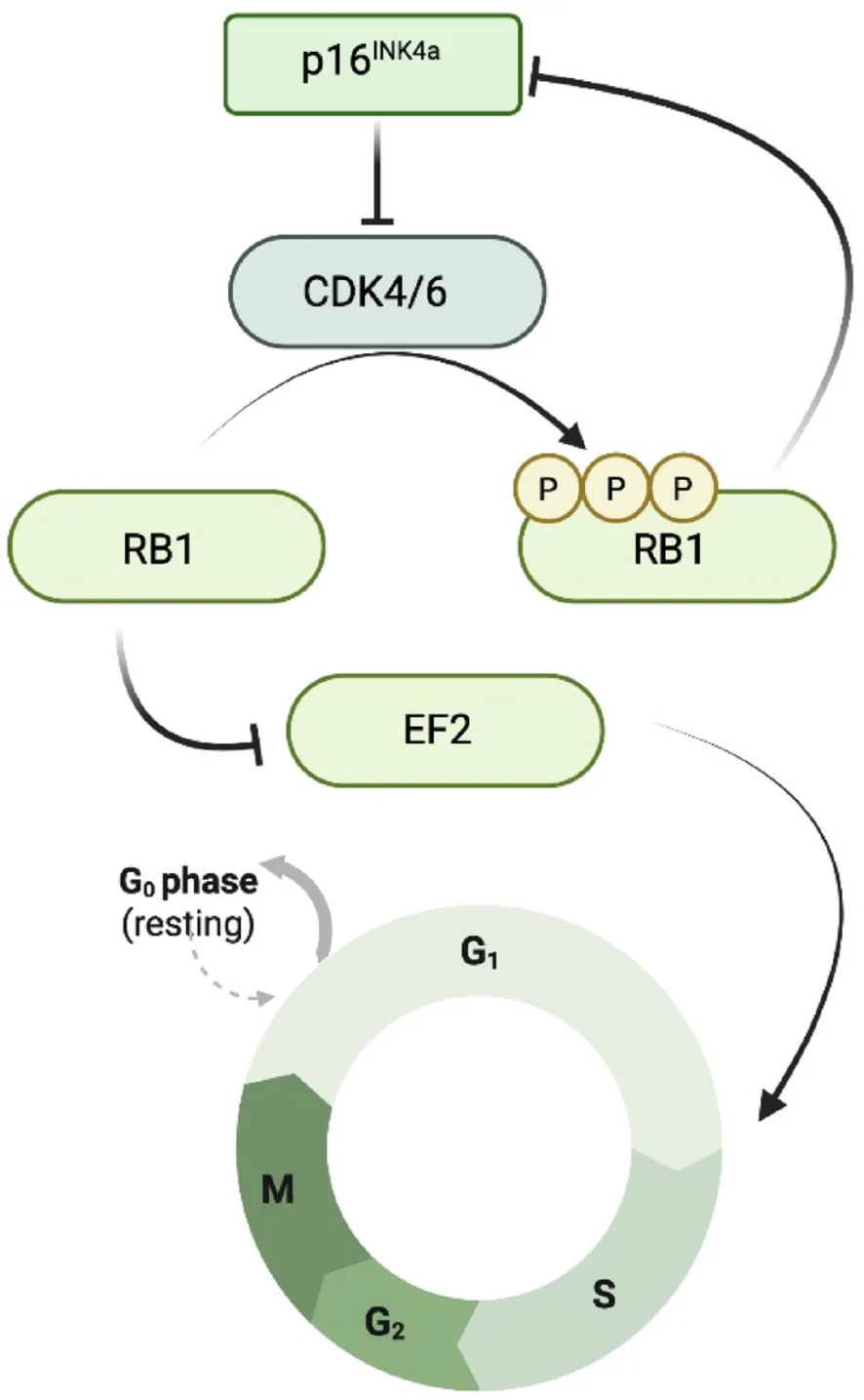
Schematic overview of p16 signaling interaction with CDK4, CDK6 and RB1 in Meningioma (Figure created with Biorender©)

*CDKN2A* plays a critical role in cell cycle progression, cellular senescence, apoptosis and is frequently altered in metastatic cancer [17, 18]. Its protein product p16^−INK4a^ binds directly to cyclin-dependent kinases CDK4 and CDK6, preventing phosphorylation of the retinoblastoma protein (Rb) (Fig. 1). Under resting conditions, E2F is sequestered by RB1. Phosphorylation of RB1 leads to dissociation of the RB1–E2F complex, allowing E2F to become transcriptionally active and initiate a comprehensive transcriptional program that drives cell-cycle progression into S phase [19]. Through the inhibition of this process, p16^−INK4a^ leads to persistence of the inactive RB1-E2F complex and thereby inactivates several proliferation-associated genes and causes cell cycle arrest in the G1 phase of tumor cells [20].

According to this regulatory mechanism, reduced p16 expression is expected to result in increased RB1 phosphorylation and, consequently, enhanced cellular proliferation. However, the expression and functional status of proteins within the p16–CDK–pRB1 pathway can be affected by multiple molecular mechanisms, with genetic alterations of *CDKN2A* representing only one of several potential determinants. *CDKN2A* alterations in meningiomas can be caused by homozygous or heterozygous gene deletions, promoter methylation, specific somatic mutations or covalent transcriptional or post-transcriptional modifications [21–23]. *CDKN2A*-associated cell-cycle deregulation is frequently seen in aggressive meningiomas and is strongly associated with cell proliferation and tumor recurrence [24].

Expression of p16 has been evaluated as a surrogate marker for CDKN2A alterations in several meningioma cohorts [15, 25, 26]. In this context absence of p16 expression, especially in higher grade meningiomas can be observed in cases with CDKN2A homozygous deletion [25, 26] However the lack of p16 protein expression in meningiomas cannot be attributed to *CDKN2A* gene inactivation alone [27]. Still, in lower grade meningiomas absence of p16 immunoreactivity is a frequently observed phenomenon and commonly attributed to low cell cycle activity [26]. Most studies in meningeal tumors have examined the correlation of p16 expression with various known *CDKN2A* alterations. Yet, there is neither much information regarding the prognostic role of p16 and other CDK pathway-related biomarkers in meningioma nor on the influence of clinical confounding factors. One study reported an association between reduced p16 expression and overexpression of CDK6 and pRB1 in 67 atypical meningiomas selected by WHO 2007 classification criteria, but statistical power was limited due to the small number of patients with tumor recurrence[28]. We therefore analyzed the frequency and prognostic value of the immunohistochemical expression of p16, CDK4, CDK6 and pRB1 (phosphorylated at S780) in a large retrospective meningioma cohort and compared their expression status in a multivariate model including established prognostic clinicopathological factors.

## Materials and methods

### Patient cohort and clinical data

Patients who were surgically treated for a meningioma in our institution between July 2003 and March 2017 were considered for inclusion. The following clinical data were collected by reviewing electronic treatment records including imaging data: histological diagnosis, WHO CNS grade, gender, age at diagnosis, tumor status (primary or recurrent meningioma), presence or absence of hereditary syndrome neurofibromatosis type 2 (NF2), tumor localization, extent of surgical resection (classified according to Simpson[29]), adjuvant radiotherapy and time to recurrence. The median follow up was 131.3 months ranging from 67.5 to 231.4 months. MIB-1 proliferation values, quantified via automated digital image analysis, were retrieved from a previous study [30]. Only cases where patient consent and tumor tissue were available, clinical records were complete and immunohistochemical staining was conclusive were included in this study. Consequently, 1751 meningiomas met the aforementioned criteria and were included in the final analysis.

### Construction of tissue microarrays and immunohistochemical staining

**Fig. 2:**
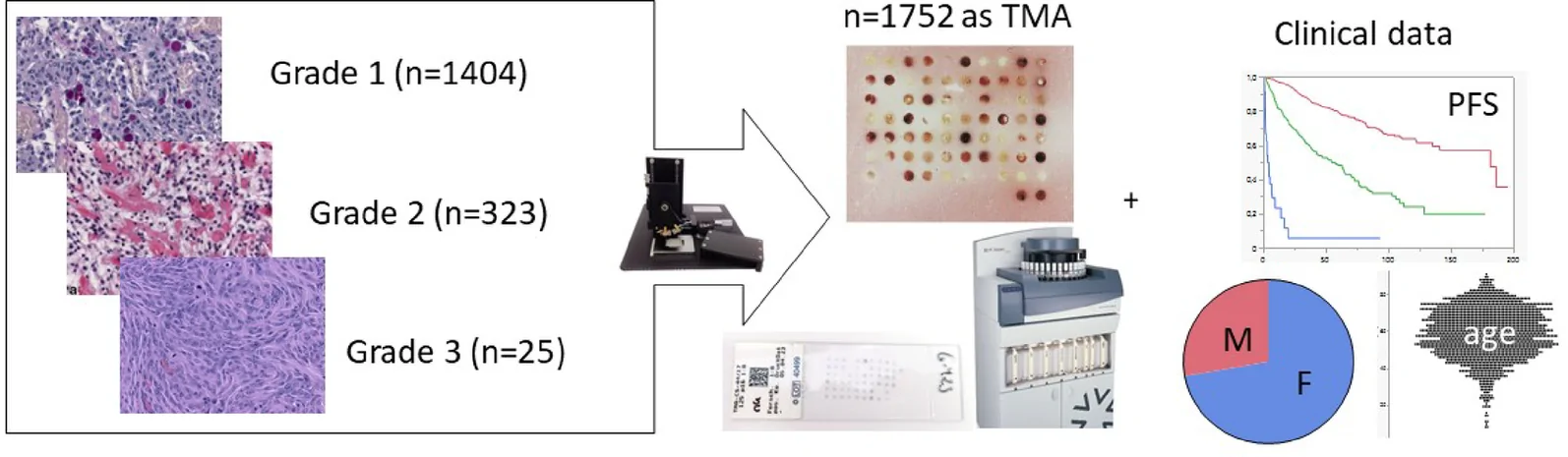
Workflow of this retrospective single-institutional study: 1751 meningiomas as tissue microarray (TMA) blocks were immunohistochemically stained for p16, CDK4, CDK6 and pRB1 and results were correlated to WHO CNS-grade, gender, age, recurrent tumor, brain invasion, MIB1, resection status and adjuvant RT. Significant PFS results from univariate analysis were included in Cox proportional hazard analysis

Formalin-fixed and paraffin-embedded (FFPE) tumor tissue samples from the archive of the Department of Neuropathology were used to construct tissue microarrays (TMA). Hematoxylin and eosin stains of the donor blocks were reviewed by an experienced neuropathologist and representative tumor areas were selected. Two sample cylinders measuring 1 mm in diameter were extracted from FFPE tumor samples using a tissue microarrayer (Beecher Instruments, Sun Prairie, Wisconsin, USA). Extracted cylinders were transferred to recipient paraffin blocks aligned in a checkerboard pattern and sealed for 5 min at 40 °C. The TMA blocks (*n *= 56 total) were cut with a microtome, producing 4 μm slices that were dried at 80° for 15 min. Immunohistochemical staining for p16 (1:2 dilution of ready-to-use Cintec p16, Roche, Basel, CH) was performed with a Ventana BenchMark immunostainer (Ventana Medical Systems, Tucson, Arizona, USA) using the OptiView immunohistochemical staining methodology (Workflow depicted in Fig. 2). Heat Induced Epitope Retrieval consisted of cell conditioner CC1 pretreatment for 32 min. The primary antibody was incubated at 37 °C for 32 min. Antigen–antibody reaction was visualized using the Ventana OptiView Universal DAB Detection kit (OptiView Linker 8 min, HRP Multimer 8 min, H_2_0_2_/DAB 8 min, Copper 4 min). All slides were then counterstained with hematoxylin for 2 min. Following conditions were used for CDK4: 1:750 dilution, CC2 pretreatment for 32 min, incubation for 32 min at 37 °C, for CDK6: 1:250 dilution, CC1 pretreatment for 64 min, incubation for 64 min at 37 °C, and for pRB1: 1:1000 dilution, CC1 pretreatment for 32 min, incubation for 32 min at room temperature. Here, the "p" in p16 merely refers to its molecular weight of 16 kDa; it is not a probe for a phosphorylated protein, unlike the phospho-specific antibody p-Rb (S780), which targets the protein only when phosphorylated at that specific site. To minimize the risk of dephosphorylation all FFPE specimens had a standardized fixation window of 12 to 24 h.

### Evaluation of antibody staining

**Fig. 3:**
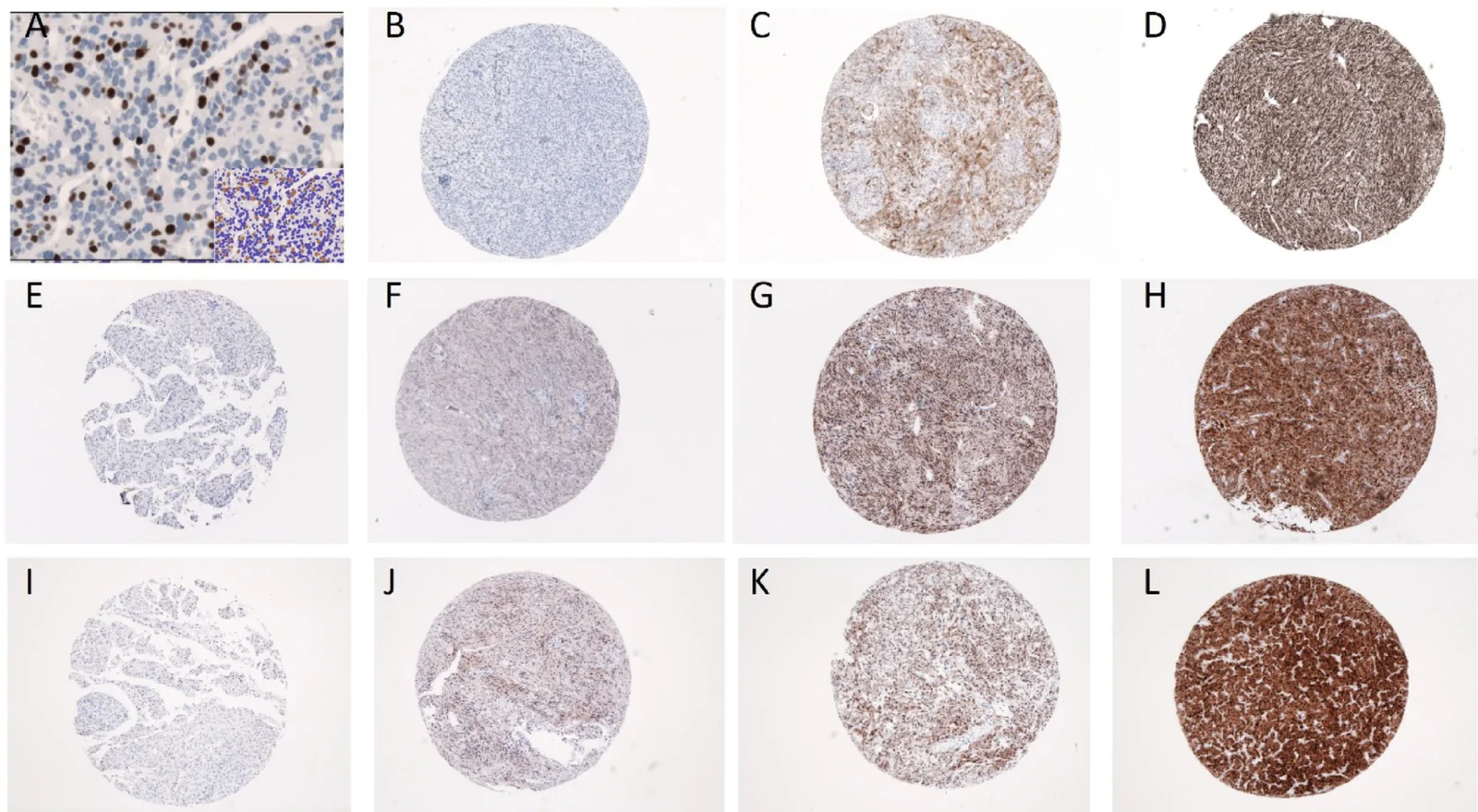
Representative immunohistochemistry stains of 1000 µm sized tissue microarray punches. Top row: **A** Higher magnification (× 200) showing nuclear pRB1 staining in a meningioma. The corresponding inset shows the matching image recognition from automated analysis using ImmunoRatio. **B** absence of p16 expression in tumor cells (score 0), **C** moderate p16 expression in 60% of the tumor cells (score 1) and **D** strong expression in all tumor cells (score 2). Middle row: Expression of CDK4 intensity scores **E** score 0, negative; **F** score 1, weak **G** score 2, moderate and **H** score 3, strong. Bottom row: Expression of CDK6 intensity scores **I** score 0, negative **J** score 1, weak **K** score 2, moderate and **L** score 3, strong

Tumor cells were evaluated for cytoplasmic and nuclear staining for p16, CDK4 and CDK6 by two independent researchers and in cases of divergence, the mean score was applied. For pRB1 only nuclear staining was analyzed by automated digital assessment. Inflammatory cells or endothelial cells were not evaluated. Stained pRB1 samples were photographed for each tumor, and automated calculation of nuclear positivity (examples are shown in supplementary Fig. 1) was performed with the ImageJ software (Version 1.51j8, NIH, Bethesda, 342 MD, USA) and the plugins Bio-Formats (Release 5.4.1; Open Microscopy Environment, 343 Madison, NJ, USA) and ImmunoRatio (Version 1.0c, Institute of Biomedical Technology, University of Tampere, Finland). For p16 two positive expression patterns were observed and a reproducible three-tiered immunohistochemical score was applied to match this pattern (Score 0: samples with less than 1% positive tumor cells or fully absent staining, score 1: heterogenous expression or focal positivity up to 75% positive tumor cells and score 2: showing diffuse homogeneous cytoplasmic staining for p16 of at least 75% of tumor cells in at least one of the two sample cylinders, Fig. 3B–D). For CDK4 and CDK6 which were homogenously expressed, combined cytoplasmic and nuclear scoring was applied as follows: 0: negative, 1: weak expression, 2 moderate expression, 3 strong expression (Fig. 3 E–H and Fig. 3 I-L respectively). After statistical exploration, scores for p16 were then dichotomized and samples with a score of ≥ 1were regarded as positive and score 0–0.75 as negative similar to interpretation methods established in cervical intraepithelial neoplasia [31]. CDK4 and CDK6 were dichotomized with 0–1: negative and > 1: positive. For pRB1, a classification and regression-tree determined cutoff was applied (scores ≥ 1.4 were considered positive). A Glioblastoma specimen controlled for absence of *CDKN2A* homozygous deletion and p16 overexpression served as positive control. Normal human brain cortex, leptomeninges and vascular endothelia served as negative control.

### CDKN2A/B Status analysis

We assessed the CDKN2A/B status in 231 cases. MethylationEPIC v2.0 BeadChip arrays were performed on genomic DNA extracted from FFPE material of human meningioma samples at the Microarray Core Facility at the DKFZ, Heidelberg, Germany. Processing of arrays was performed in R (v4.4.2) with the SeSAMe package (v1.24.0). CNV profiles from DNA methylation array data were generated using the conumee2 package (v2.1.2), using 121 normal CNS tissue samples as a reference line. As we have encountered high levels of tumor content in meningioma samples (average of 77%), we considered the following absolute log2 ratios of total signal intensities as cutoffs to call copy number variants for individual genomic loci: < 0.3 (no CNV), > 0.3 to 0.8 (heterozygous CNV), and > 0.8 (homozygous CNV).

### Statistical analysis

The Pearson’s chi-squared test was applied for contingency analyses of antibody expression and clinical factors. Binary logistic regression was used to assess factors associated with p16, CDK4, CDK6 and pRB1 expression in cases where information on recurrence or progression was available. Afterwards p-values were adjusted with Bonferroni-Holm correction. Kaplan–Meier analysis was conducted for univariate prognostic assessment using the log-rank test. The multivariate analysis was done applying the Cox proportional hazard analysis and the Wald test. A significance level of α < 0.05 was applied. For the definition of a prognostic cutoff for MIB1 proliferation, p16, CDK4, CDK6, pRB1 indices and patient age at diagnosis a classification and regression tree (CART) analysis was performed. For additional inclusion of the CDK-pathway into the prognostic model, three groups were defined: intact (p16 positive, pRB1 below the CART-specific cut-off), disrupted (p16 negative, pRB1 above the CART-specific cut-off) and discordant (miscellaneous). The three options were tested in a multivariate model against the discordant variant and in a second step included into the multivariate model including the parameters above. The software JMP® Statistical Discovery Software, version 18.2 (Cary, NC: SAS Institute Inc.; 1989) and IBM Statistics SPSS, version 30.0.0 (IBM, Armonk, NC, USA) was used for the statistical analysis. For visualization GraphPad Prism version 10.6.1 (GraphPad Software, Boston, USA) were used. The study was approved by the institutional Clinical Ethics Committee (Project numbers: 618/2014BO2 and 191/2021BO2).

## Results

The overall cohort consisted of 1751 meningiomas (1269 female and 482 male; mean age: 58.9 years; range: 8.2–90.9 years, including 38 patients below the age of 18 years). Immunohistochemical results were available for p16 in 1751 samples, for CDK4 in 1751 samples, CDK6 in 1751 samples and for pRB1 in 1706 samples. Reasons for exclusion after staining were either insufficient tumor tissue on available TMA slides or focal staining failure (especially for pRB1).

### Expression of p16 in tumor samples

Of 1751 meningioma samples 261 tumors were immunopositive for p16 (14.9%). Staining was mainly cytoplasmic but additional nuclear p16 positivity was frequently observed in tumor cells exhibiting cytoplasmic staining. Inflammatory cells and tumor vessels were negative for p16.

The distribution of p16 expression according to clinical and histopathological characteristics is summarized in Table 1. When examining tissue of recurrent meningiomas at the time of investigation, the frequency of p16 immunopositivity was significantly higher than in tissue resected from primary cases (35.2% vs. 13.4%, *p* < 0.0001). The proportion of p16 immunopositivity was significantly higher in CNS WHO grade 2 (27.5%) and grade 3 tumors (25.0%) than in CNS WHO grade 1 tumors (11.8%, *p* < 0.0001).

**Table 1:** Distribution of p16 expression according to clinical and histopathological characteristics (Pearson’s chi-squared test)

	*N* (%)	p16 expression (*N*, %)		*p*-value	Holm-adjusted *p*-value
		Positive	Negative		
Gender					
Female	1269 (72.5)	155 (12.2)	1114 (87.8)	< 0.0001*	0.0011*
Male	482 (27.5)	106 (22.0)	376 (78.0)		
Age					
≥ 45.49	1454 (83.1)	222 (15.3)	1232 (84.7)	0.3394	1.00
< 45.49	297 (16.9)	39 (13.1)	258 (86.9)		
Location					
Skull base	819 (51.2)	86 (10.5)	733 (89.5)	< 0.0001*	0.0011*
Convexity/falx	622 (38.9)	132 (21.2)	490 (78.8)		
Spinal	158 (9.9)	35 (22.1)	123 (77.8)		
Prim/Rec					
Primary	1423 (89.0)	191 (13.4)	1232 (86.6)	< 0.0001*	0.0011*
Recurrent	176 (11.0)	62 (35.2)	114 (64.8)		
NF2					
Yes	36 (2.2)	6 (16.7)	30 (83.3)	0.8891	0.8891
No	1563 (97.8)	247 (15.8)	1316 (84.2)		
Simpson grade					
I/II/III	1123 (71.7)	173 (15.4)	950 (84.6)	0.4698	1.0
IV/V	444 (28.3)	75 (16.9)	369 (83.1)		
CNS WHO grading					
1	1403 (80.1)	166 (11.8)	1237 (88.2)	< 0.0001*	0.0011*
2	318 (18.2)	88 (27.7)	230 (72.3)		
3	30 (1.7)	7 (23.3)	23 (76.7)		
Histology					
1					
Angiomatous	36 (2.1)	1 (2.8)	35 (97.2)	< 0.0001*	0.0011*
Fibroblastic	134 (7.6)	19 (14.2)	115 (85.8)		
Lymphocyte rich	2 (0.1)	0 (-)	2 (100)		
Meningothelial	863 (49.3)	105 (12.2)	758 (87.8)		
Metaplastic	21 (1.2)	3 (14.3)	18 (85.7)		
Microcystic	34 (1.9)	2 (5.9)	32 (94.1)		
Psammomatous	63 (3.6)	11 (17.5)	52 (82.5)		
Secretory	52 (3.0)	6 (11.5)	46 (88.5)		
Transitional	182 (10.4)	15 (8.2)	167 (91.8)		
NOS	16 (0.9)	4 (25.0)	12 (75.0)		
2					
Atypical	286 (16.3)	87 (30.4)	199 (69.6)		
Chordoid	32 (1.8)	1 (3.1)	31 (96.9)		
Clear Cell	0 (-)	0 (-)	0 (-)		
3					
Anaplastic	24 (1.4)	6 (25.0)	18 (75.0)		
Papillary	0 (-)	0 (-)	0 (-)		
Rhabdoid	6 (0.3)	1 (16.7)	5 (83.3)		
MIB1					
≥ 6.1%	122 (7.6)	44 (36.1)	78 (63.9)	< 0.0001*	0.0011*
< 6.1%	1475 (92.4)	206 (13.9)	1269 (86.1)		
Tumor recurrence					
Yes	329 (23.1)	78 (23.7)	251 (76.3)	< 0.0001*	0.0011*
No	1097 (76.9)	155 (14.1)	942 (85.9)		
Adjuvant RT					
Yes	72 (4.5)	39 (54.2)	33 (45.8)	< 0.0001*	0.0011*
No	1502 (93.9)	210 (14.0)	1292 (86.0)		

Additionally, a highly significant gender difference was observed, with a higher rate of p16-positive meningiomas in males than in females (22.0% vs. 12.2%, *p *= 0.0001). Spinal and convexity meningiomas had a higher rate of p16 positivity (22.1% and 21.2%, respectively) compared to tumors located in the skull base (10.5%, *p *= 0.0001). Within the cohort of primary tumors, the rate of p16 positivity was significantly higher in tissue stained from primary cases that later showed recurrence or tumor progression during follow-up (23.7% vs. 14.1%, *p* < 0.0001). Tumors with proliferation above the CART-specific MIB1 cutoff of 6.1% showed a significantly higher rate of p16 positivity (36.1% vs.13.9%, *p* < 0.0001). Tumors of patients, who received adjuvant radiotherapy after resection, which were in total *n *= 72 with 55% of them being classified as CNS WHO Grade 2 or 3, showed a significantly higher rate of p16 expression (54.2% compared to 13.9%, *p* < 0.0001). No staining difference of p16 was observed using the age cutoff at 45.59 years. Similarly, p16 expression was comparable between sporadic tumors (15.8%) and NF2-associated germline tumors (16.7%) as well as between completely (Simpson grade I-III: 15.4%) and incompletely resected tumors (Simpson grade IV/V: 16.9%).

### Expression of CDK4 and CDK6 in tumor samples

CDK4 expression was present in 735 (41.9%) samples and homogeneous throughout tissue punches with varying staining intensities reflected in the expression scores (Supplementary Table S1). Similarly, CDK6 expression was observed in 739 (42.2%) samples. Detailed data on CDK4 and CDK6 distribution are provided in Supplementary Tables S2 and S3. There was a significant difference in CDK4 expression regarding gender (male: 46.3% vs. female: 40.3%, *p* < 0.025) and tumor status at the time of resection, with tissue from recurrent tumors showing higher positivity than tissue from primary tumors (57.4% vs. 42.6%, *p* < 0.0001). Convexity/falx and skull base locations had significantly higher rates of CDK4-positive tumors than spinal locations (51.7% and 47.2% vs. 32.9%, *p* < 0.0001). For CDK6 positivity, higher rates were found in intracranial tumors compared to spinal meningiomas with a clear predominance in the convexity or falx (convexity/falx: 56.4%, skull base: 35.8% vs. spinal 28.5%, *p* < 0.0001). While the rate of CDK4-positive tumors was higher in CNS WHO grade 2 (52.8%) and grade 3 (50.0%) compared to WHO grade 1 tumors (39.3%, *p* < 0.0001), the inverse was observed for CDK6: higher rates of CDK6 positivity were found in CNS WHO grade 1 (41.1%) and grade 2 (48.4%) tumors than in grade 3 tumors (28.6%, *p *=  0.0180).

Tissue from primary meningiomas that later demonstrated recurrence or progression during follow-up exhibited a higher rate of CDK4 positivity (50.1% vs. 43.8%, *p* < 0.0442). Samples from patients, who later received adjuvant radiotherapy showed significantly higher rates of CDK4 positivity (70.8% vs. 42.7%, *p* < 0.0001). Tumors with a MIB-1 index above the CART-specific cutoff of 6.1% were also more frequently CDK4 positive (61.5% vs. 42.6%, *p* < 0.0001). Conversely, no significant differences in CDK6 positivity were observed regarding MIB1 index, gender, primary versus recurrent tumors, history of progression afterwards or later application of adjuvant radiotherapy. In relation to extent of resection, age and germline NF2 status neither CDK4 nor CDK6 positivity showed a significant difference.

After adjustment for Bonferroni-Holm correction all p-values remained significant except for gender and later tumor recurrence in respect to CDK4 status and WHO grading in CDK6 status.

### Expression of pRB1 in tumor samples

For pRB1 expression, a CART-specific cutoff at 1.4% was determined and the distribution of expression and associations with different clinical and histopathological parameters are shown in Table 2. Of the 1706 meningioma samples that evaluated 589 (34.5%) demonstrated high pRB1 expression. Tumors from male patients displayed significantly higher pRB1 expression than those from female patients (42.9% vs. 31.3% *p* < 0.0001). Tissue from recurrent tumors showed high pRB1 expression more frequently than tissue from primary tumors (59.6% vs. 42.9%, *p* < 0.0001). An initial difference was observed regarding the extent of resection, with high pRB1 expression in 40.5% of Simpson grade IV/V resections compared to 33.9% in Simpson grade I-III resected tumors (*p *= 0.0158), however after adjustment for Bonferroni-Holm correction this parameter did not remain significant.

**Table 2:** Distribution of pRB1 cutoff expression according to clinical and histopathological characteristics

	*N* (%)	pRB1 cutoff expression (*N*, %)		*p*-value	Holm-adjusted *p*-value
		High	Low		
Gender					
Female	1235 (72.4)	387 (31.3)	848 (68.7)	< 0.0001*	0.0011*
Male	471 (27.6)	202 (42.9)	269 (57.1)		
Age					
≥ 45.49	1417 (83.1)	484 (34.2)	933 (65.8)	0.4784	1.0
< 45.49	289 (16.9)	105 (36.3)	184 (63.7)		
Location					
Skull base	799 (51.2)	276 (34.5)	523 (65.5)	0.4028	1.0
Convexity/falx	608 (38.9)	221 (36.4)	387 (63.6)		
Spinal	155 (9.9)	62 (40.0)	93 (60.0)		
Prim/Rec					
Primary	1391 (89.0)	457 (32.8)	934 (67.2)	< 0.0001*	0.0011*
Recurrent	171 (11.0)	102 (59.6)	69 (40.4)		
NF2					
Yes	36 (2.3)	12 (33.3)	24 (66.7)	0.756	1.0
No	1526 (97.7)	547 (35.9)	979 (64.1)		
Simpson grade					
I/II/III	1099 (71.8)	373 (33.9)	726 (66.1)	0.0158 *	0.0790
IV/V	432 (28.2)	175 (40.5)	257 (59.5)		
CNS WHO grading					
1	1369 (80.4)	421 (30.8)	948 (69.2)	< 0.0001*	0.0011*
2	311 (18.2)	150 (48.2)	161 (51.8)		
3	26 (1.4)	18 (69.2)	8 (30.8)		
Histology					
1					
Angiomatous	35 (2.1)	16 (45.7)	19 (54.3)	< 0.0001*	0.0011*
Fibroblastic	133 (7.8)	27 (20.3)	106 (79.7)		
Lymphocyte rich	2 (0.1)	1 (50.0)	1 (50.0)		
Meningothelial	842 (49.4)	268 (31.8)	574 (68.2)		
Metaplastic	20 (1.2)	4 (20.0)	16 (80.0)		
Microcystic	34 (2.0)	14 (41.2)	20 (58.8)		
Psammomatous	60 (3.5)	16 (26.7)	44 (73.3)		
Secretory	52 (3.1)	28 (53.8)	24 (46.2)		
Transitional	175 (10.3)	37 (21.1)	138 (78.9)		
NOS	16 (0.9)	10 (62.5)	6 (37.5)		
2					
Atypical	280 (16.4)	136 (48.6)	144 (51.4)		
Chordoid	31 (1.8)	14 (45.2)	17 (54.8)		
Clear Cell	0 (-)	0 (-)	0 (-)		
3					
Anaplastic	20 (1.2)	13 (65.0)	7 (35.0)		
Papillary	0 (-)	0 (-)	0 (-)		
Rhabdoid	6 (0.3)	5 (83.3)	1 (16.7)		
MIB1					
≥ 6.1%	118 (7.6)	77 (65.3)	41 (34.7)	< 0.0001*	0.0011*
< 6.1%	1442 (92.4)	474 (32.9)	968 (67.1)		
Tumor recurrence					
Yes	317 (22.7)	162 (51.1)	155 (48.9)	< 0.0001*	0.0011*
No	1077 (77.3)	336 (31.2)	741 (68.8)		
Adjuvant RT					
Yes	67 (4.4)	49 (73.1)	18 (26.9)	< 0.0001*	0.0011*
No	1470 (95.6)	498 (33.9)	972 (66.1)		

Samples of CNS WHO grade 3 and grade 2 tumors more frequently presented high pRB1 expression levels (66.7% and 48.6%, respectively) than grade 1 tumors (30.8%, *p* < 0.0001).

A highly significant difference in the distribution of high pRB1 expression was noted in tumors with a MIB1 index above the before via CART-analysis determined cutoff of 6.1% (65.3%) compared to those below it (32.9%, *p* < 0.0001). Furthermore, high pRB1 expression was more common in tissue samples from patients with a history of later recurrence or later need of adjuvant radiotherapy (51.1% and 73.1%, respectively) compared to tumor samples from patients without adjuvant radiotherapy or a stable follow up (31.2% and 33.9%, respectively; *p* < 0.0001 for both parameters). No significant difference in pRB1 expression was seen regarding age, germline NF2 status and tumor location.

### Correlation analysis for p16, CDK4, CDK6 and pRB1

Spearman’s rank correlation was performed to assess the relationships between of p16 expression and the other histological markers CDK4, CDK6 and pRB1 (Supplementary Fig. S2). A highly significant but moderate correlation was found between p16 positivity and positive staining for CDK4 (Spearman’s ρ=0.302, *p* < 0.001).

Only a weak correlation between p16 and CDK6 positivity was found (ρ=0.087, *p* < 0.001), while a relevant correlation between CDK4 and CDK6 positivity was observed (ρ=0.41, *p* < 0.001). For high pRB1 expression a correlation with CDK4 and CDK6 could only be observed on a weak level (ρ=0.114 and ρ=0.096 respectively, for both *p* < 0.001). A weak correlation (ρ=0.155, *p* < 0.001) was found between p16 positivity and pRB1 high expression.

### Correlation analysis for p16 and pRB1 with CDKN2A/B status

We performed Spearmans’ rank correlation between p16 immunostaining and CDKN2A/B status, which was available in 231 cases. In 7 cases homozygous CDKN2A/B deletion was observed. All 7 cases were negative for p16 immunostaining. No correlation between CDKN2A/B hemizygous deletion and p16 immunoreactivity could be found (Spearmans-Rho − 0.077, *p *= 0.265). We additionally performed a Spearmans’ rank correlation between pRB1 immunostaining above the cut-off and presence of a homozygous CDKN2A/B deletion, where we found a signigicant correlation of pRB staining above the cut-off with presence of homozygous CDKN2A/B status (Spearmans-Rho 0.232, *p* < 0.001**). CDKN2A/B homozygous deleted cases are color coded as green datapoints in Supplementary Fig. S2.

### Binary regression of clinical factors with p16, CDK4, CDK6 and pRB1

The multivariate assessment of clinical variables that potentially influence the p16, CDK4, CDK6 and pRB1 expression was examined with a nominal logistic regression analysis. Details of the nominal logistic fit and odds ratios for p16 and pRB1 are displayed in Tables 3 and 4; results for CDK4 and CDK6 are shown in Supplementary Tables S4 and S5.

**Table 3:** Nominal logistic regression of factors associated with p16 expression

	Odds ratio (95%CI)	*p*-value (Prob>Chisq)
Male gender	1.58 (1.15–2.19)	0.005*
Age > 45.9	0.96 (0.64–1.45)	0.852
Location		
Intracranial versus spinal	0.38 (0.24–0.60)	< 0.001*
Primary versus recurrent meningioma	2.46 (1.61–3.77)	< 0.001*
NF2 versus sporadic	1.24 (0.47–3.26)	0.664
CNS WHO grading		
1 versus 2	1.84 (1.27–2.66)	< 0.001*
2 versus 3	0.76 (0.23–2.51)	0.657
MIB1 > 6.1%	1.63 (0.99–2.70)	0.053
CDK4 expression	3.91 (2.80–5.46)	< 0.001*
CDK6 expression	1.18 (0.86–1.61)	0.299
pRB1 expression > 1.4	1.44 (1.05–1.96)	0.022*

**Table 4:** Binary logistic regression of factors associated with high pRB1expression

	Odds ratio (95%CI)	*p*-value (Prob>Chisq)
Male gender	1.43 (1.11–1.83)	0.005*
Age > 45.9	1.11 (0.82–1.49)	0.496
Location		
Intracranial versus spinal	0.66 (0.46–0.96)	0.027*
Primary versus recurrent meningioma	2.29 (1.58–3.32)	0.001*
NF2 versus sporadic	0.93 (0.44–1.98)	0.525
CNS WHO grading		
1 versus 2	1.35 (1.00–1.83)	0.049*
2 versus 3	0.69 (0.25–2.66)	0.735
MIB1 > 6.1%	2.79 (1.77–4.39)	< 0.001*
CDK4 expression	1.05 (0.83–1.33)	0.710
p16 expression	1.44 (1.06–1.96)	0.020*
CDK6 expression	1.24 (0.98–1.57)	0.071

Male gender, recurrent tumor status at the time of staining and CNS WHO grade 2, CDK4 immunopositivity, and high pRB1 expression were independently associated with higher immunopositivity rates for p16 expression. Conversely, spinal localization was associated with lower p16 expression.

CDK4 immunopositivity was associated with intracranial tumor localization, and immunopositivity for both CDK6 and p16, while CDK6 immunopositivity was associated with female gender, intracranial tumor localization, primary tumor status and CDK4 positivity.

For pRB1 expression above the cutoff independent associations were found with male gender, age above the CART-specific cutoff of 45.59 years, spinal tumor localization, primary tumor status, WHO grade 2, higher MIB1 index and the presence of p16 and CDK6 immunostaining.

When evaluating the joint impact of the marker expression in a combined CDK-pathway model, we identified three functional groups with distinct independent risks for tumor recurrence. Compared to the discordant phenotype, an intact CDK pathway was associated with a protective effect (HR = 0.60, p = 0.032), whereas a disrupted pathway was an independent predictor of accelerated recurrence (HR = 1.55, *p* < 0.001; Supplementary Table S6).

### Recurrence and progression-free survival in relation to clinical factors and p16, CDK4, CDK6 and pRB1

Information on recurrence or progression after the sampling determined with follow-up imaging was available for 1426/1751 cases (81.4%) with a median follow-up of 131.3 months ranging from 67.5 to 231.4 months. Tumor recurrence was recorded in 329 cases (23.1%). Univariate analyses of standard clinical factors associated with the rate of tumor recurrence are shown in Table 5.

**Table 5:** Univariate analysis of factors associated with tumor recurrence (Pearson’s chi-squared test, two-sided)

	*N* (%)	Tumor recurrence (N, %)		*p*-value	Holm-adjusted *p*-value
		Yes	No		
Gender					
Female	1041 (73.0)	193 (18.5)	848 (81.5)	0.001*	0.013*
Male	385 (27.0)	136 (35.3)	249 (64.7)		
Age					
≥ 45.49	1176 (82.5)	259 (22.0)	917 (78.0)	0.042*	0.294
< 45.49	250 (17.5)	70 (28.0)	180 (72.0)		
Location					
Skull base	740 (51.9)	177 (23.9)	563 (76.1)	0.001*	0.013*
Convexity/falx	558 (39.1)	140 (25.1)	418 (74.9)		
Spinal	128 (9.0)	12 (9.4)	116 (90.6)		
NF2					
Yes	35 (2.5)	3 (8.6)	32 (91.4)	0.039*	0.294
No	1391 (97.5)	326 (23.4)	1065 (76.6)		
Simpson grade					
1/2/3	996 (71.3)	149 (15.0)	847 (85.0)	< 0.001*	0.013*
4/5	400 (28.7)	174 (43.5)	226 (56.5)		
CNS WHO grading					
1	1147 (80.4)	189 (16.5)	958 (83.5)	< 0.001*	0.013*
2	253 (17.8)	117 (45.9)	138 (54.1)		
3	26 (1.8)	25 (96.2)	1(3.8)		
Histology				< 0.001*	0.013*
1					
Angiomatous	30 (2.1)	2 (6.7)	28 (93.3)		
Fibroblastic	110 (7.7)	9 (8.2)	101 (91.8)		
Lymphocyte rich	2 (0.1)	0 (-)	2 (100)		
Meningothelial	716 (50.2)	139 (19.4)	577 (80.6)		
Metaplastic	20 (1.4)	0 (-)	20 (100.0)		
Microcystic	28 (2.0)	5 (17.9)	23 (82.1)		
Psammomatous	48 (3.4)	3 (6.2)	45 (93.8)		
Secretory	42 (2.9)	6 (14.3)	36 (85.7)		
Transitional	139 (9.7)	21 (15.1)	118 (84.9)		
NOS	12 (0.9)	4 (33.3)	8 (66.7)		
2					
Atypical	225 (15.8)	100 (44.4)	125 (55.6)		
Chordoid	28 (2.0)	14 (50.0)	14 (50.0)		
Clear Cell	0 (-)	0 (-)	0 (-)		
3					
Anaplastic	21 (1.5)	21 (100.0)	0 (-)		
Papillary	0 (-)	0 (-)	0 (-)		
Rhabdoid	5 (0.3)	4 (80.0)	1 (20.0)		
Adjuvant radiotherapy					
Yes	76 (5.3)	24 (31.6)	52 (68.4)	0.072	0.432
No	1347 (94.7)	305 (22.6)	1042 (77.4)		
MIB1					
≤ 6.1% or missing	1268 (92.1)	255 (20.1)	1013 (79.9)	0.001*	0.013*
> 6.1%	109 (7.9)	63 (57.8)	46 (42.2)		
P16 expression					
Yes	233 (16.4)	78 (33.5)	155 (66.5)	0.001*	0.013*
No	1192 (83.6)	251 (21.1)	941 (78.9)		
CDK4 expression				0.051	0.306
Yes	1064 (74.6)	259 (24.3)	805 (75.7)		
No	362 (25.4)	70 (19.3)	292 (80.7)		
CDK6 expression				0.905	0.905
Yes	960 (67.5)	223 (23.2)	737 (76.8)		
No	462 (32.5)	106 (22.9)	356 (77.1)		
pRB1				0.001*	0.013*
> 1.4%	498 (35.8)	162 (32.5)	336 (67.5)		
< 1.4% or missing	895 (64.2)	155 (17.3)	740 (82.7)		

*significance level of α < 0.05 are shown in bold)

Patients whose tumors demonstrated positive p16 expression had a significantly higher risk of later recurrence (33.5% vs. 21.1%, *p* < 0.001). Similarly, pRB1 expression above the cutoff of 1.4% was associated with higher rates of later tumor recurrence (32.5% vs. 17.3%, *p* < 0.001). No association between tumor recurrence and expression of CDK4 and CDK6 was observed within the follow-up cohort.

A significantly lower recurrence rate was associated with complete resection (Simpson grade I-III), female gender, spinal tumor localization and lower CNS WHO tumor grade (*p* < 0.001 for all mentioned variables). Conversely, higher rates of tumor recurrence were seen in tumors with an expression level for MIB1 reaching 6.1% or higher (*p *= 0.001) and in patients younger than the CART-determined age cutoff at 45.59 (*p *= 0.042). Patients with germline NF2-associated tumors showed a lower recurrence rate (8.6%) than those with sporadic meningiomas (23.4%, *p *= 0.039). No association was found between the rate of tumor recurrence and a history of adjuvant radiotherapy. After adjustment according to Bonferroni-Holm correction all p-values except those for age and germline NF2-status remained significant.

### Cox proportional hazard of progression-free survival

**Fig. 4:**
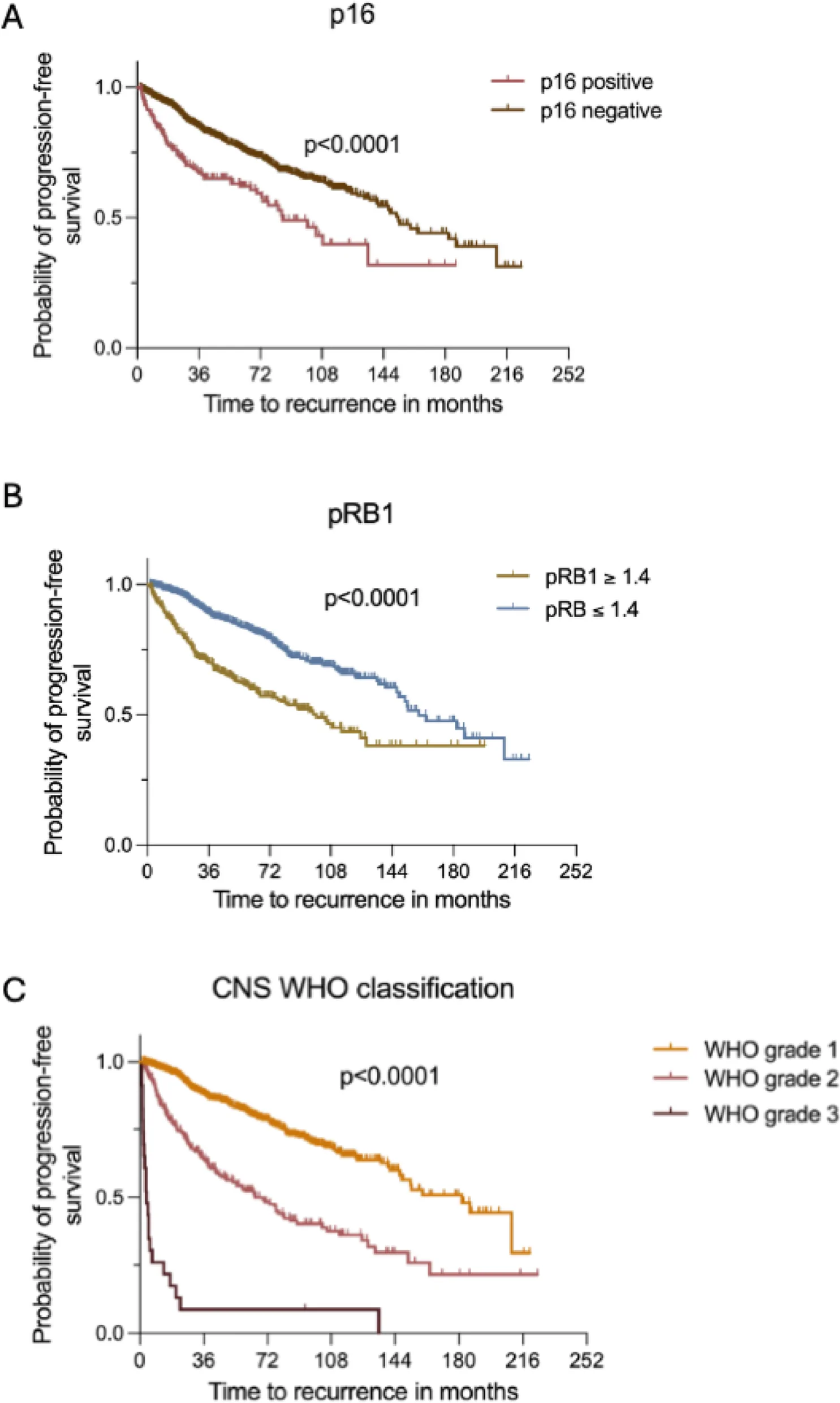
Kaplan–Meier graphs for progression-free survival in 1426 meningioma samples stratified by **A** p16 expression status, **B** pRB1 expression level and **C** tumor WHO CNS grade

Kaplan–Meier graphs of progression-free survival for WHO CNS grade, p16 and pRB1 tumor status are shown in Fig. 4. CDK4 and CDK6 curves are shown in Supplementary Fig. S3. CDK6 expression did not show a difference in progression free survival. Stratification by WHO CNS grade revealed that pRB1 expression retained strong prognostic significance in grade 2 and 3 tumors (Log-rank *p* < 0.0001), whereas p16 expression did not (Supplementary Fig. S4).

For multivariate Cox proportional hazard of progression free survival, all significant parameters in the univariate analysis assessing the risk for recurrence together with the application of adjuvant radiotherapy were included in a multivariate model (Table 6 and Supplementary Table S7). For p16 expression a trend towards a higher risk of tumor progression (HR 1.08, 95%CI: 0.80–1.45) was observed, although this did not reach statistical significance (*p *= 0.608). In contrast, pRB1 expression above the cutoff was highly significantly associated with an increased risk of tumor recurrence (HR 2.15, 95%CI: 1.69–2.72, *p* < 0.001). Other highly significant factors associated with increased risk of tumor progression included higher WHO CNS grade, an MIB-1 index above the cutoff of 6.1%, and incomplete resection (Simpson Grade > III). The administration of post-operative adjuvant radiotherapy was highly significantly associated with lower risk of subsequent tumor recurrence (HR 0.31, 95% CI: 0.20–0.49 *p* < 0.001). Male gender was also significantly associated with an increased risk of tumor progression, while germline NF2-associated tumors showed lower rates of tumor recurrence. In the combined CDK-pathway model a highly significant difference was observed between disrupted and discordant expression patterns (*p* < 0.0001; Supplementary Fig. S5).

**Table 6:** Multivariate analysis of parameters associated with tumor recurrence

	Hazard ratio (95%CI)	*p*-value
Male gender	1.32 (1.03–1.70)	0.031*
Age > 45.59	1.20 (0.90–1.59)	0.217
Location		
Convexity/Falx versus spinal	1.66 (0.84–3.29)	0.205
Skull base versus spinal	1.18 (0.59–2.37)	0.637
Simpson grade ≤ 3	0.28 (0.22–0.35)	< 0.001*
NF2 versus sporadic	0.27 (0.09–0.88)	0.029*
CNS WHO grading		
1 versus 2	2.96 (2.24–3.91)	< 0.001*
1 versus 3	24.18 (12.86–45.46)	< 0.001*
MIB1 > 6.1	2.26 (1.58–3.24)	< 0.001*
P16 expression	1.08 (0.80–1.45)	0.608
pRB1 > 1.4	2.15 (1.69–2.72)	< 0.001*
Adjuvant radiotherapy	0.31 (0.19–0.49)	< 0.001*

## Discussion

Determining predictive factors for the risk of meningioma recurrence is under intense ongoing research. *CDKN2A/B* homozygous deletion is currently one of two established molecular markers that have been incorporated into the fifth edition of the CNS WHO brain tumor classification for meningiomas [5, 32]. Furthermore, other genetic alterations of *CDKN2A* have been shown to be associated with aggressive tumor behavior in meningioma [33]

Previous studies examining the role of p16 expression as an immunohistochemical surrogate marker for *CDKN2A* have provided mixed results[24, 27, 28, 34]. In a large multi-omics analysis Wang et al. showed a correlation between elevated *CDKN2A* mRNA levels, p16 protein expression and elevated CDK4 on both mRNA and protein levels, as well as either deficient or hyperphosphorylated RB1, suggesting elevated cell cycle activity [35].

Discrepancies of p16 protein expression frequency and *CDKN2A* homozygous deletion alone in meningiomas are commonly observed [25]. This is not surprising because p16 expression is also influenced by hotspot mutations in the *CDKN2A* gene and the methylation of the *CDKN2A* promoter and can be regulated by histone modifications such as histone H3 Lys27 trimethylation [13, 21, 36]. Also diverging methylation patterns across tumor alleles were provided as possible explanation [24]. A high sensitivity, but low specificity of p16 as a surrogate marker for CDKN2A gene status has also been reported in astrocytic brain tumors [37]. Consistently, in our subset analysis, all cases with homozygous CDKN2A/B deletions showed a total loss of p16 immunoreactivity, whereas hemizygous deletions or intact status did not correlate with p16 protein expression levels. Therefore, the aim in our current study was not to correlate p16, CDK4, CDK6 or pRB1 with CDKN2A molecular status or methylation classifier data. Instead, we sought information regarding the prognostic role of p16 and the associated signaling pathway members CDK4, CDK6 and pRB1 immunohistochemistry in a comprehensive multivariate model. This approach also reflects the common practice in neuropathology where molecular risk assessment is restricted to cases with a specific clinical history.

We performed our analysis on a large and well-established meningioma cohort with high-quality clinical and follow-up data including cases that were classified as CNS WHO grade 3 based on their *TERT* and *CDKN2A* status [11]. We analyzed a subset of 1751 meningiomas and found p16 expression in 14.9% of examined tumors. This rate is lower than the 70% to >90% positivity reported in the recent literature evaluating p16 loss as a surrogate for molecular deletions [25] where any minor staining is considered positive. In contrast, our study specifically evaluated p16 overexpression by utilizing a strict three-tiered system. Furthermore, our protocol strictly focused on tumor cells only, deliberately excluding p16-positive non-neoplastic bystander cells—such as infiltrating macrophages and fibroblastic components—which can otherwise artificially inflate rates. Finally, because our large unselected cohort is heavily dominated by indolent grade 1 meningiomas without active cell-cycle progression, a lower overall rate of p16 overexpression is not surprising. We found significantly lower rates of p16 positivity in meningiomas located at the skull base compared to lesions within the convexity/falx or spine, which might be linked to different genetic profiles of tumors located at the skull base, which had recently been described [38]. Our finding that p16 expression increases alongside higher CNS tumor grades reflects this elevated cell cycle activity and cellular stress, tracking significantly with recurrence, high MIB-1 (≥ 6.1%), and post-radiotherapy status, thereby supporting our dichotomized approach. This paradoxical upregulation of a tumor suppressor in aggressive tumors is a known feedback phenomenon [39]. When high-grade tumors undergo intense oncogenic stress or downstream pathway disruptions (such as RB1 inactivation), normal negative feedback loops fail, prompting a compensatory hyper-expression of non-functional p16 protein. Thus, elevated p16 IHC in advanced meningiomas serves as a surrogate for proliferative and microenvironmental stress rather than functional tumor suppression.

The significantly higher rates of p16 and pRB1 expression observed in male patients in our cohort align with the established clinical observation that meningiomas in men often exhibit more aggressive biological behavior than those in women. Similar male-predominant p16 overexpression has been documented in other malignancies, such as head and neck squamous cell carcinomas and sinonasal cancers [40]. A study by Terzi et al. reported p16 overexpression via immunohistochemistry in 16/39 (41%) WHO grade II tumors classified according to the previous 4th revised WHO 2007 edition and statistical association with event-free survival, also Tang et al. reported p16 immunoreactivity in 32/43 WHO Grade 2 and 3 meningioma, while only 5/14 WHO Grade 1 meningioma showed p16 expression with a high sensitivity but low specificity for prediction of CDKN2A/B homozygous deletion [26, 34]. Also, Ozkizilkaya et al. as well as Zschernack et al. reported high rates of p16 immunostaining with 41/43 and 3/9 positive cases in highly selective tumor samples with underlying CDKN2A/B alterations and stated p16 negativity as a predictor for CDKN2A/B deletion [25, 41].

Compared to these cohorts our p16 immunoreactivity rate is markedly lower. Still, this effect can be explained due to the unselective and substantially larger patient cohort, reflecting real world data with a much higher proportion of CNS WHO Grade 1 tumors.

Using the CNS WHO 2000 classification Korshunov et al. reported a lower frequency of p16-positive tumors in meningioma recurrences but did not find a statistical association with clinical outcome [27]. Since then, the grading and classification of meningiomas has significantly changed due brain invasion and recently introduced molecular factors [12, 32]. Simon et al. examined p16 by Western Blot and reported the absence of p16 protein in 16/46 (35%) meningiomas without correlation to tumor recurrence [42]. Because the studies by Korshunov et al. (38% p16 positive) and Kim et al. (65% p16 positive) evaluated only nuclear p16 expression, results from our study and the data presented by Terzi et al. are not directly comparable [27, 28]. Recent data have confirmed that cytoplasmic p16 expression in cancer is indeed relevant for tumor progression, while nuclear p16 is not [43, 44].

A previous study employing the CNS WHO 2016 classification analyzed 140 meningiomas and associated pRB1 hyperphosphorylation at the S780 site with decreased progression-free survival [45]. To the best of our knowledge, this is the first large cohort study to use digital assessment for evaluating pRB1 immunohistochemical expression in meningiomas. Although our CART-determined pRB1 cutoff of 1.4% is specific to this cohort and requires multicenter validation, it can be easily and objectively replicated using standard digital image analysis software rather than casual visual assessment. High pRB1 expression was found in 34.5% of the 1706 analyzed meningiomas and was significantly associated with a higher CNS WHO grade, an elevated MIB-1 index, recurrent tumor status, and a history of tumor recurrence, emphasizing its prognostic role.

Despite the limited data on pRB1 expression in meningioma, a pan-cancer molecular TGCA-based analysis on the RB1 pathway found alterations in the RB1 axis in about 30% of all cases and established an integrated CDK4/6-RB1-expression signature profile, which was associated with a worse prognosis especially in tumors with low basal proliferation [46]. However, the mentioned work only focused on genetic analyses, which are not always in accordance with immunohistochemical protein expression and due to economic reasons are not broadly available. This is underlined by the findings of Burns et al., who not only pointed out that pRB1 immunohistochemistry results differed from the *RB1* gene status in glioblastoma, but also showed that there is a complex interaction within the p16/CDK4/CDK6/RB1-axis in this tumor entity with unknown prognostic relevance [47].

Positive staining for CDK4 was spotted in 41.9% and CDK6 expression in 42.2% in 1751 tumors investigated. Both were not independent predictors in multivariate models in relation to clinical factors, suggesting that these markers of the CDK pathway are less informative than p16 and pRB1. This is the first study to systematically investigate the expression of all of the above-mentioned markers together in meningiomas. A correlation of p16 with all other markers CDK4, CDK6 and pRB1 was determined, suggesting a complex interaction within the entire signaling pathway with possible prognostic relevance for meningiomas.

While univariate analysis showed an association between p16 immunopositivity or elevated pRB1 expression with both the rate and risk of tumor recurrence and to unfavorable progression-free survival, the picture is not complete without including all other established prognostic influences. The multivariate analysis did not demonstrate an association of p16 expression and shorter PFS, clearly indicating, that p16 might not be an independent negative prognostic factor. There is however a tight correlation between high proliferation and CDK pathway activation in our cohort; tumors with a MIB-1 index ≥ 6.1% showed significantly higher p16 positivity (36.1% vs. 13.9%). This mirrors phenomena in other hyper-proliferative tissues—such as cervical lesions—where intense oncogenic stress triggers a compensatory, non-functional upregulation of cell-cycle regulators [48].

In contrast high pRB1 expression was identified as a highly significant negative prognostic factor in the multivariate analysis, alongside higher WHO CNS grade, subtotal resection, an elevated MIB-1 index, male gender and no adjuvant radiation therapy. Sporadic meningiomas also had a significantly higher risk of recurrence compared to NF2-associated tumors. While the latter well-established clinical prognostic factors serve as a proof of concept for the statistical model, high expression of pRB1 stands out as the most reliable histopathological marker of the examined components of the CDK signaling complex.

Some authors have reported a reciprocal relationship between phosphorylated Rb (p-Rb) and p16^−INK4a^ expression suggesting that tumors with intact pRb show low levels of p16^−INK4a^ and vice versa [22, 49]. However other studies propose a more complex and dynamic interaction between the two markers [46, 50]. The association between p16 positive staining and high levels of pRB1 might thereby explain the univariate prognostic effect of p16, which is lost when other established prognostic factors are integrated into a multivariate analysis. Our observation that both, p16 and pRB1 are upregulated in a subset of meningiomas—rather than the reciprocal relationship described in some other cancer types—suggests a complex synergy of cell-cycle dysregulation in unfavorable cases resulting from a feedback loop failure [51]. In this context, the elevated pRB1 expression potentially overrides upstream p16 signaling and thus might explain why it is the sole independent prognostic factor of the assessed cell cycle regulators in our multivariate model. Reflecting this biological complexity, our combined CDK-pathway model demonstrated strong utility by identifying three functional groups with distinct independent risks, where an intact pathway expression pattern proved protective and a disrupted status independently predicted accelerated tumor recurrence.

To date there is no established chemotherapy regimen for meningiomas. Despite surgical resection and post-operative radiotherapy, patients with WHO CNS grade 2 or grade 3 tumors have high rates of progression and eventual disease-associated mortality [7]. Tumors with reduced or absent p16 expression are generally considered more sensitive to the cyclin-dependent kinases 4 and 6 inhibitor palbociclib. Recent experimental data using meningioma cell lines suggest that palbociclib monotherapy as well as the combination of palbociclib and radiation extends the survival of mice bearing orthotopic p16-deficient meningioma xenografts [52]. Furthermore, a benefit regarding progression free survival in patients with recurrent meningioma CNS WHO grade 2 and 3 treated with the CDK-inhibitor abemaciclib has been described [53]. These results imply that CDK inhibitors might be a promising therapeutic option for selected meningioma patients. Therefore, systematic analyses for the predictive and prognostic relevance of the individual components in the CDK pathway are crucial.

Our data indicates that pRB1 has superior prognostic utility compared to p16, showing a highly significant independent association with shortened progression-free survival in our large meningioma cohort. The persistent prognostic significance of pRB1 in WHO grade 2 and 3 tumors highlights its potential as a valuable candidate for routine immunohistochemical stratification in neuropathological practice.

Conclusions drawn from this study require validation in prospective cohorts and should also be correlated with molecular data including methylation, proteomics and metabolomics.

The main limitation of our study is its retrospective single-center design. We examined a large consecutive cohort of surgically treated meningiomas. Consequently, asymptomatic tumors that are managed conservatively are underrepresented. Furthermore, the interpretation of immunohistochemical stainings may vary between observers despite extensive neuropathological experience with meningioma specimens in our institute. Furthermore, the analyzed tissue samples (tissue microarray cylinders), may not be always representative for the entire tumor tissue due to potential intratumor heterogeneity. Despite our maximal efforts to standardize tissue handling, pRB1 remains highly sensitive to fixation delays and pre-analytical artifacts due to its nature as a phospho-specific epitope. p16-negative cases may contain samples without an active cell cycle as well as cases with true loss of p16 due to homozygous deletion, complicating the interpretation of negative staining in lower-grade meningiomas. Despite these limitations pRB1 serves as a more reliable biomarker in neuropathology routine because its alteration directly drives cell cycle progression and according to our data more accurately correlates with aggressive tumor behavior and poor clinical outcomes.

## Conclusions

In conclusion, our data in a large meningioma cohort indicate that pRB1 expression is an independent prognostic factor associated with shorter progression-free survival. The interaction within the associated p16/CDK4/CDK6/RB1 signaling axis remains complex, but suggests that the prognostic impact of p16 in meningioma is likely attributed to its involvement in this essential cell cycle pathway, where pRB1 might act as the key downstream effector. Further validation of pRB1 as a prognostic marker in large multicentric biomolecular datasets is warranted to correlate these findings with clinical outcomes.

## Supplementary Information

Below is the link to the electronic supplementary material.

Supplementary Material 1.

Supplementary Material 2.

Supplementary Material 3.

## Data Availability

The dataset is available from the corresponding author upon reasonable request.
