# Supplementary material for: The superior prognostic role of pRB1 over p16 immunohistochemistry as a biomarker in meningiomas
Source: Acta Neuropathol Commun. 2026 Sep 3;14:186. doi: 10.1186/s40478-026-02419-3 (PMC13548670; doi:10.1186/s40478-026-02419-3)

S1: Exemplary pRB1 immunostains with expression below cutoff (left column) and above cutoff (right column)

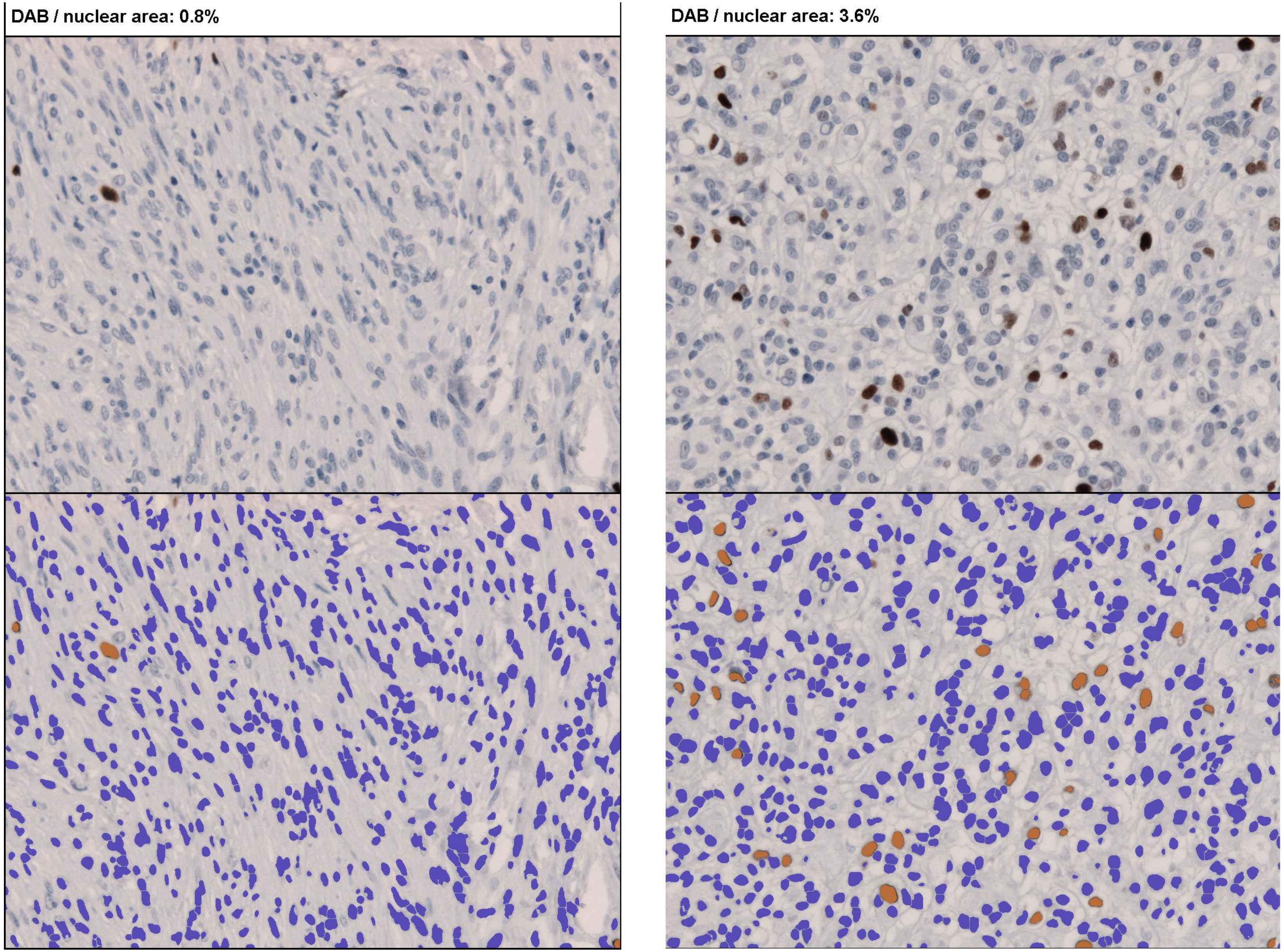

S2: Graphs for pairwise correlation of individual datapoints for p16, CDK4, CDK6 and pRB1

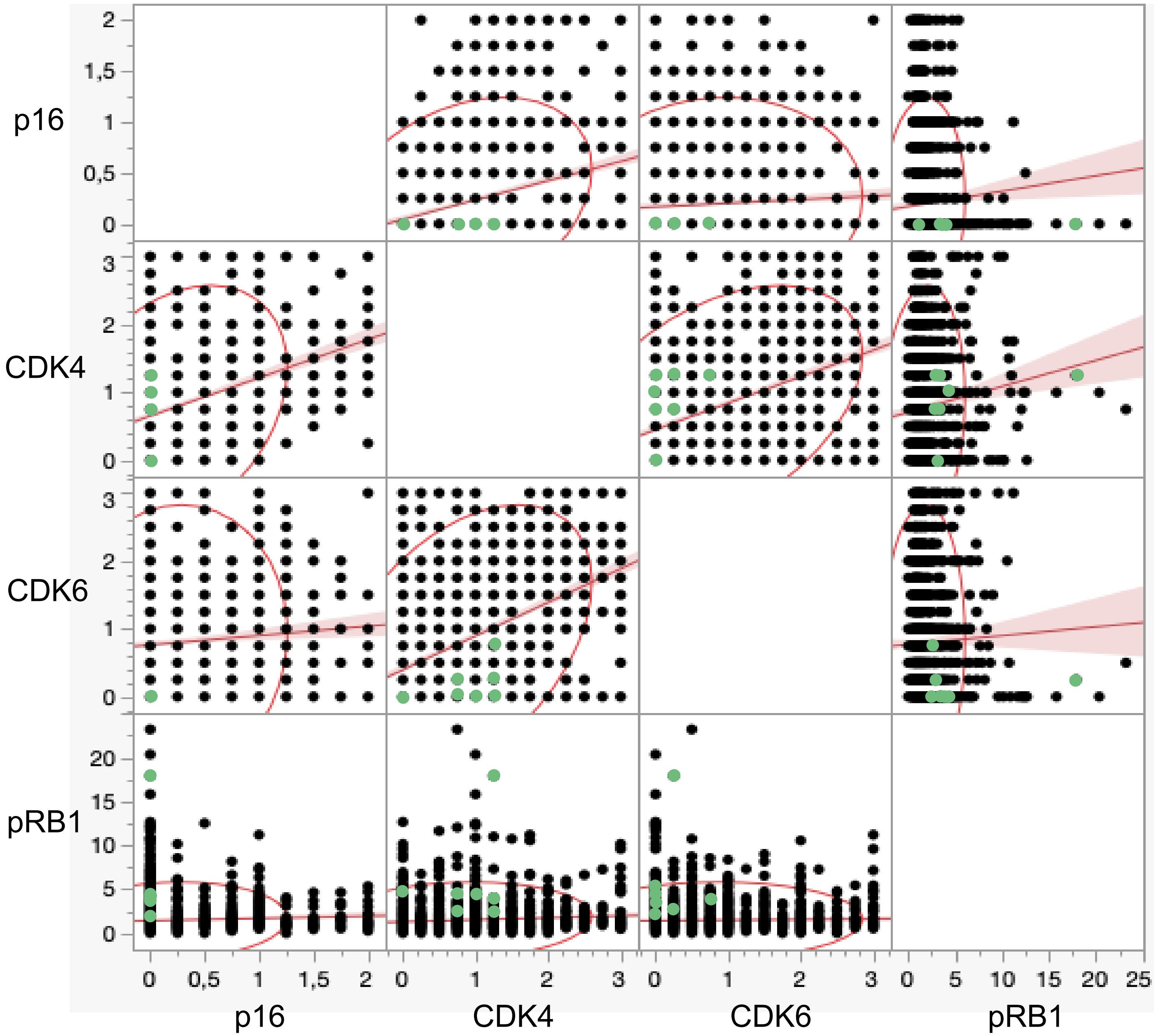

S3: Kaplan-Meier analysis for CDK4 (top) and CDK6 (bottom)

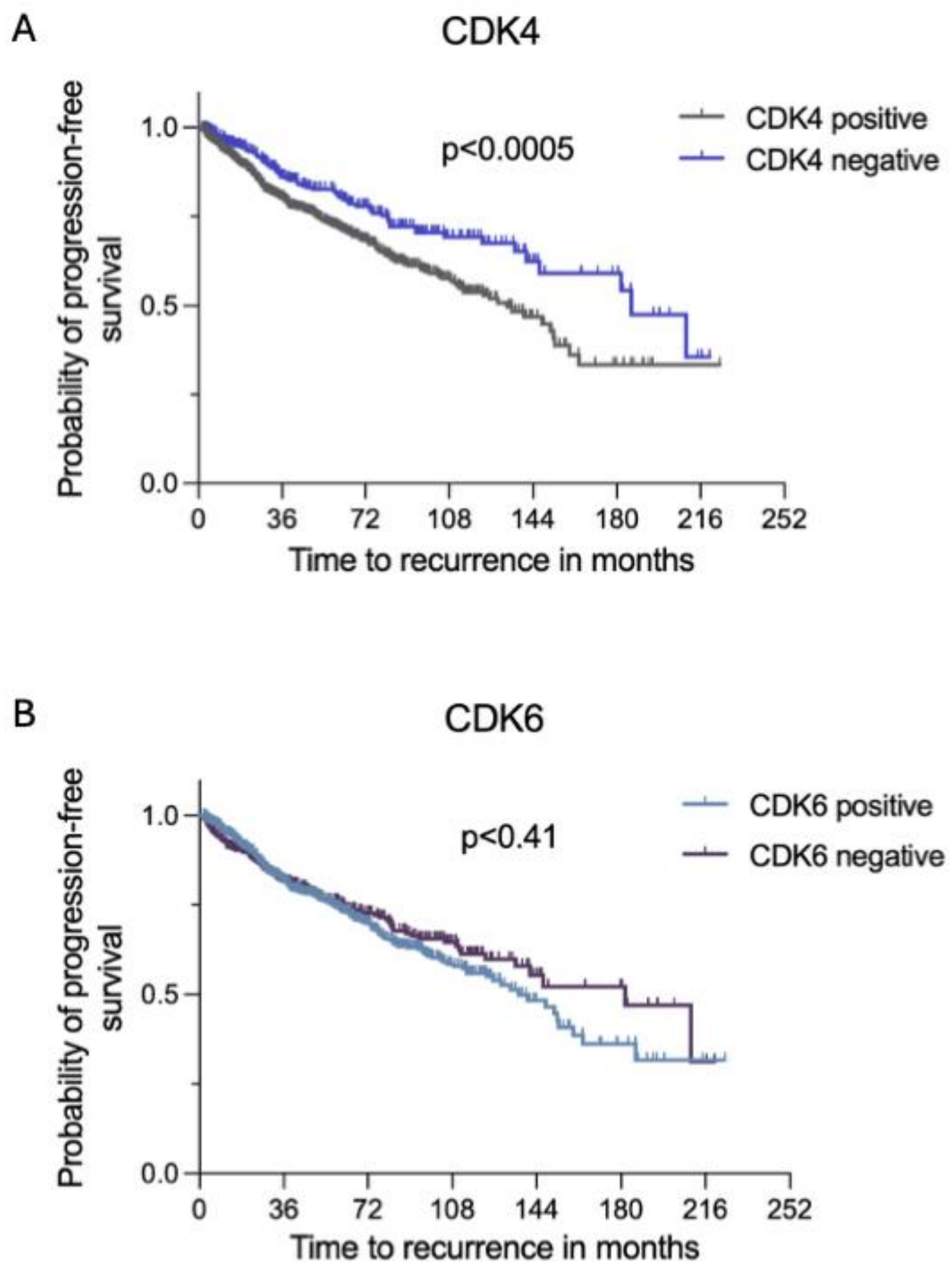

S4: Kaplan-Meier subgroup analysis for p16 (left column) and pRB1 (right column) separated by WHO CNS grade 1 (top) vs grade 2 and 3 (bottom)

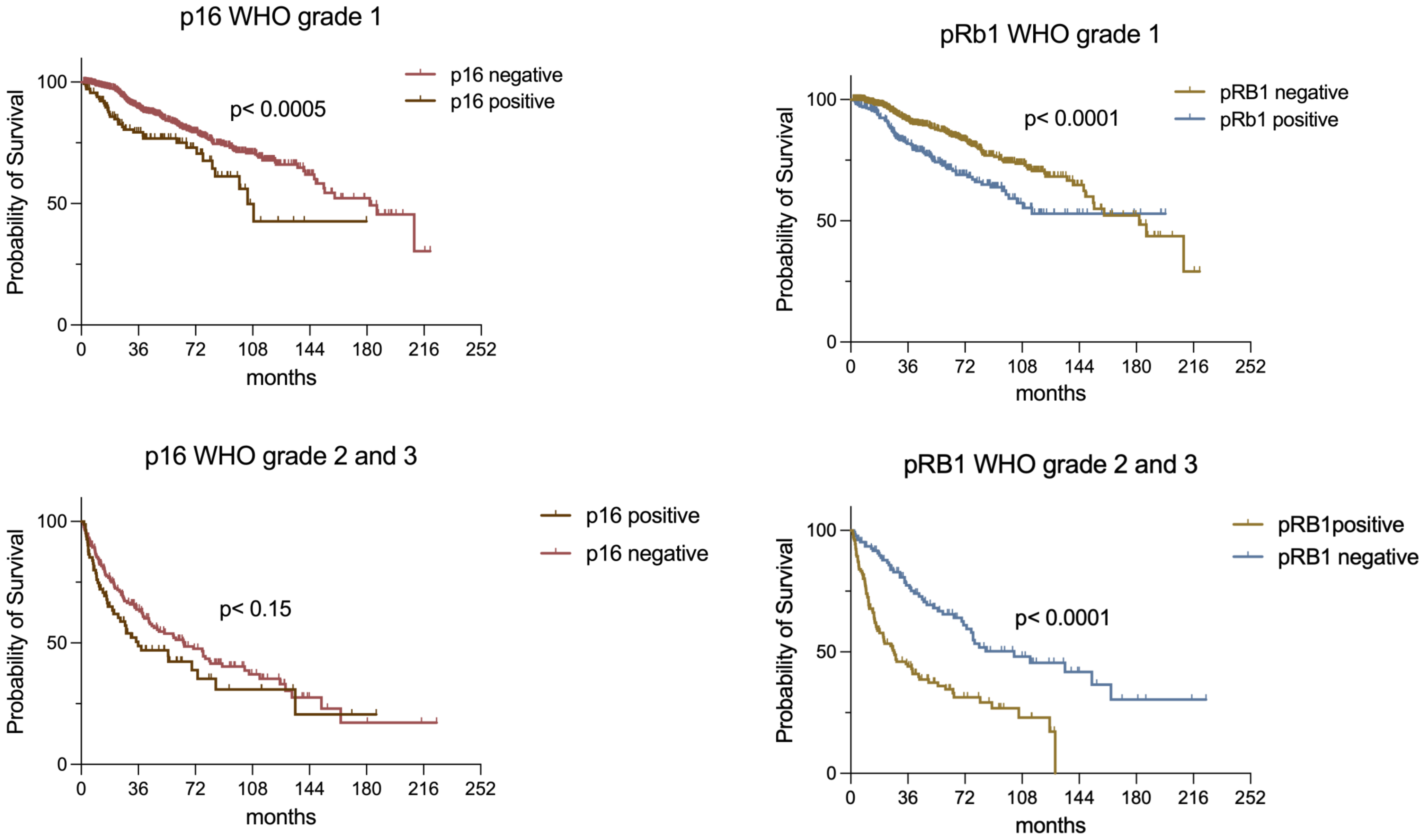

S5: Kaplan-Meier subgroup analysis for CDK-pathway combined groups: Intact (p16 positive / pRB1 low), Disrupted (p16 negative / pRB1 high), and Discordant (miscellaneous combination).

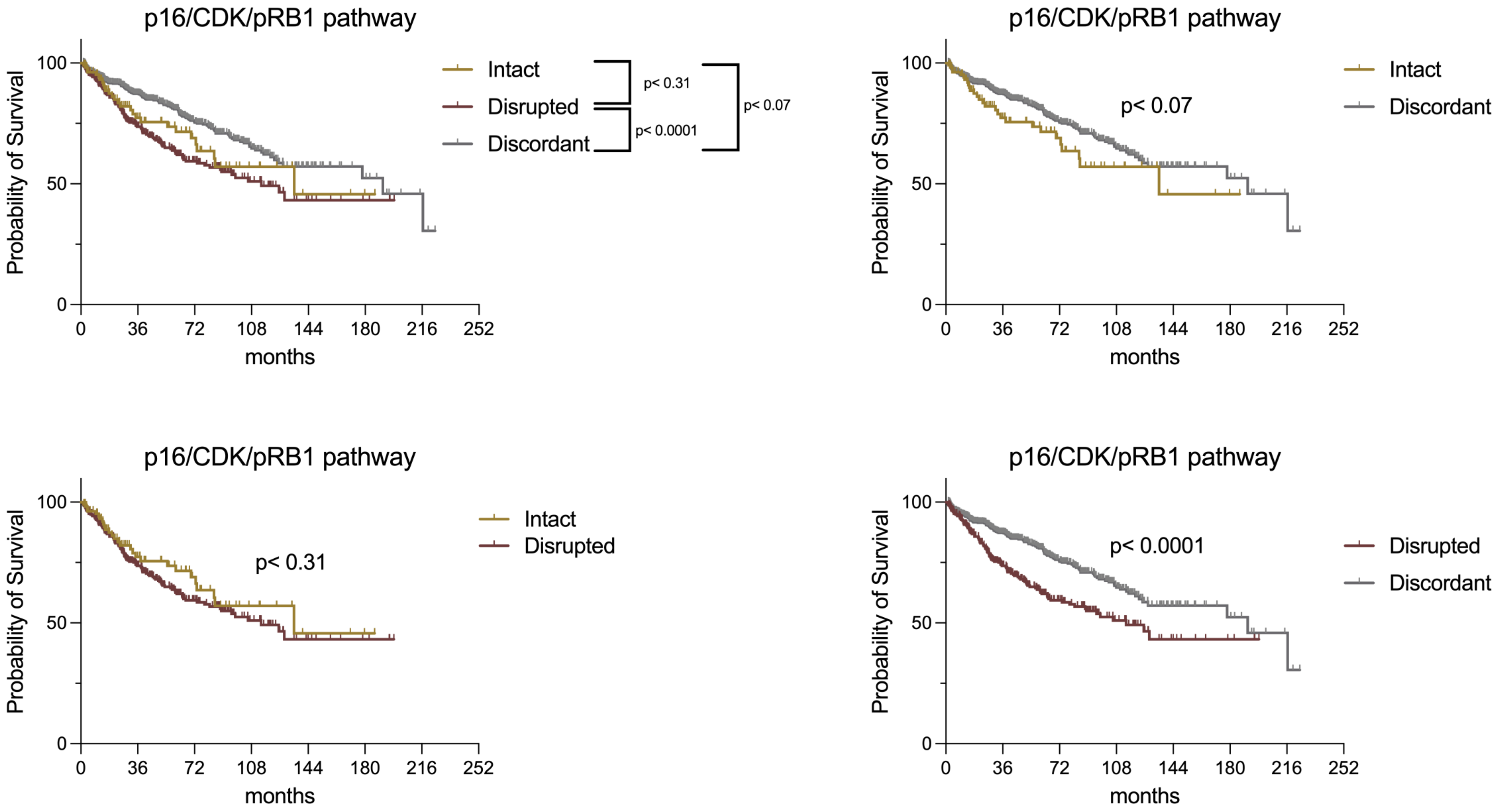

Supplement: Supplementary file 1 [file 40478_2026_2419_MOESM3_ESM.pdf]
