# Supplementary material for: The superior prognostic role of pRB1 over p16 immunohistochemistry as a biomarker in meningiomas
Source: Acta Neuropathol Commun. 2026 Sep 3;14:186. doi: 10.1186/s40478-026-02419-3 (PMC13548670; doi:10.1186/s40478-026-02419-3)
Supplement: Supplementary file 2 [file 40478_2026_2419_MOESM1_ESM.docx]

Table S1: Distribution of staining intensity scores for p16, CDK4 and CDK6 antibodies

|  | p16 (n=1751) | CDK4 (n= 1751) | CDK6 (n= 1751) |
| --- | --- | --- | --- |
| 0 | 1405 (80.4%) | 1016 (58.0%) | 1012 (57.8%) |
| 1 | 291 (16.6%) | 531 (30.3%) | 474 (27.1%) |
| 2 | 55 (3.1%) | 179 (10.2%) | 227 (12.9%) |
| 3 | 0 | 25 (1.4%) | 38 (2.2%) |

Table S2: Distribution of CDK4 expression according to clinical and histopathological characteristics (Pearson’s chi-squared test).

|  | |  | | **CDK4 expression (N, %)** | |  | |  | |
| --- | --- | --- | --- | --- | --- | --- | --- | --- | --- |
|  | **N (%)** | | **Positive** | | **Negative** | | **p-value** | | **Holm-adjusted  p-value** |
| Gender  Female  Male  Age  >/=45.49  < 45.49  Location  Skull base  Convexity/falx  Spinal    Prim/Rec  Primary  Recurrent    NF2  Yes  No  Simpson grade  I/II/III  IV/V  CNS WHO grading  1  2  3  Histology  1  Angiomatous  Fibroblastic  Lymphocyte rich  Meningothelial  Metaplastic  Microcystic  Psammomatous  Secretory  Transitional  NOS  2  Atypical  Chordoid  Clear Cell  3  Anaplastic  Papillary  Rhabdoid    MIB1  >=6.1%  <6.1%  Tumor recurrence  Yes  No  Adjuvant RT  Yes  No | 1269 (72.4)  482 (27.5)  1454 (83.0)  297 (16.9)  819 (51.2)  622 (38.9)  158 (9.9)  1423 (89.0)  176 (11.0)  36 (2.2)  1563 (97.8)  1123 (71.7)  444 (28.3)  1403 (80.1)  318 (18.2)  30 (1.7)  36 (2.1)  134 (7.6)  2 (0.1)  864 (49.3)  21 (1.2)  34 (1.9)  63 (3.6)  52 (3.0)  182 (10.4)  16 (0.9)  285 (16.3)  32 (1.8)  0 (-)  24 (1.4)  0 (-)  6 (0.3)  122 (7.6)  1475 (92.4)  329 (23.1)  1097 (76.9)  72 (4.5)  1502 (93.9) | | 512(40.3)  223 (46.3)  611 (42.0)  124 (41.8)  334 (40.8)  322 (51.8)  52 (32.9)  607 (42.6)  101 (57.4)  17 (47.2)  691 (44.2)  508 (45.2)  188 (42.3)  552 (39.3)  167 (52.5)  16 (53.3)  12 (33.3)  51 (38.1)  0 (-)  341 (39.5)  6 (28.6)  13 (38.2)  21 (33.3)  17 (32.7)  88 (48.3)  4 (25.0)  154 (54.0)  12 (37.5)  0 (-)  15 (62.5)  0 (-)  1 (16.7)  75 (61.5)  628 (42.6)  165 (50.2)  481 (43.8)  51 (70.8)  642 (42.7) | | 757 (59.7)  259 (53.7)  843 (58.0)  173 (58.2)  485 (59.2)  300 (48.2)  106 (67.1)  816 (57.4)  75 (42.6)  19 (52.8)  872 (55.8)  615 (54.8)  256 (57.7)  851 (60.7)  151 (47.5)  14 (46.7)  24 (66.6)  83 (61.9)  2 (100)  523 (60.5)  15 (71.4)  21 (61.8)  42 (66.7)  35 (67.3)  94 (51.7)  12 (75.0)  131 (46.0)  20 (62.5)  0 (-)  9 (37.5)  0 (-)  5 (83.3)  47 (38.5)  847 (57.4)  164 (49.8)  616 (56.2)  21 (29.2)  860 (57.3) | | 0.0250*  0.9312  <0.0001*  0.0002*  0.7190  0.2989  <0.0001*  0.0002*  <0.0001*  0.0442*  <0.0001* | | 0.125  0.9312  0.0014*  0.0014*  1.0  0.8967  0.0011*  0.0014*  0.0011*  0.1789  0.0011* |

Table S3: Distribution of CDK6 expression according to clinical and histopathological characteristics (Pearson’s chi-squared test).

|  |  | **CDK6 expression (N, %)** | |  |  |
| --- | --- | --- | --- | --- | --- |
|  | **N (%)** | **Positive** | **Negative** | **p-value** | **Holm-adjusted p-value** |
| Gender  Female  Male  Age  >/=45.49  < 45.49  Location  Skull base  Convexity/falx  Spinal    Prim/Rec  Primary  Recurrent    NF2  Yes  No  Simpson grade  I/II/III  IV/V  CNS WHO grading  1  2  3  Histology  1  Angiomatous  Fibroblastic  Lymphocyte rich  Meningothelial  Metaplastic  Microcystic  Psammomatous  Secretory  Transitional  NOS  2  Atypical  Chordoid  Clear Cell  3  Anaplastic  Papillary  Rhabdoid    MIB1  >=6.1%  <6.1%  Tumor recurrence  Yes  No  Adjuvant RT  Yes  No | 1269 (72.4)  482 (27.5)  1454 (83.0)  297 (16.9)  819 (51.2)  622 (38.9)  158 (9.9)  1423 (89.0)  176 (11.0)  36 (2.2)  1563 (97.8)  1123 (71.7)  444 (28.3)  1403 (80.1)  318 (18.2)  30 (1.7)  36 (2.1)  134 (7.6)  2 (0.1)  864 (49.3)  21 (1.2)  34 (1.9)  63 (3.6)  52 (3.0)  182 (10.4)  16 (0.9)  285 (16.3)  32 (1.8)  0 (-)  24 (1.4)  0 (-)  6 (0.3)  122 (7.6)  1475 (92.4)  329 (23.1)  1097 (76.9)  72 (4.5)  1502 (93.9) | 546 (43.0)  193 (40.0)  621 (42.7)  118 (39.7)  293 (35.8)  351 (56.4)  45 (28.5)  619 (43.5)  70 (39.8)  19 (52.8)  670 (42.9)  497 (44.3)  176 (39.6)  576 (41.1)  155 (48.7)  8 (26.7)  21 (58.3)  73 (54.5)  0 (-)  322 (37.3)  6 (28.6)  22 (64.7)  21 (33.3)  12 (23.1)  97 (53.3)  3 (18.7)  143 (50.2)  11 (34.4)  0 (-)  8 (33.3)  0 (-)  0 (-)  55 (45.0)  630 (42.7)  135 (41.0)  493 (44.9)  26 (36.1)  649 (43.2) | 723 (57.0)  289 (60.0)  833 (57.3)  179 (60.3)  526 (64.2)  271 (43.6)  113 (71.5)  804 (56.5)  106 (60.2)  17 (47.2)  893 (57.1)  626 (55.7)  268 (60.4)  827 (58.9)  163 (51.3)  22 (73.3)  15 (41.7)  61 (45.5)  2 (100)  542 (62.7)  15 (71.4)  12 (35.3)  42 (66.7)  40 (76.9)  85 (46.7)  13 (81.3)  142 (49.8)  21 (65.6)  0 (-)  16 (66.7)  0 (-)  6 (100)  67 (55.0)  845 (57.3)  194 (59.0)  604 (55.1)  46 (63.9)  853 (56.8) | 0.2587  0.3435  <0.0001*  0.3462  0.2351  0.0962  0.0180*  <0.0001*  0.6112  0.2290  0.2083 | 1.0  1.0  0.0011*  1.0  1.0  0.7696  0.1620  0.0011*  1.0  1.0  1.0 |
