# Supplementary material for: The superior prognostic role of pRB1 over p16 immunohistochemistry as a biomarker in meningiomas
Source: Acta Neuropathol Commun. 2026 Sep 3;14:186. doi: 10.1186/s40478-026-02419-3 (PMC13548670; doi:10.1186/s40478-026-02419-3)
Supplement: Supplementary file 3 [file 40478_2026_2419_MOESM2_ESM.docx]

Table S4: Nominal logistic regression of factors associated with CDK4 expression.

|  | **Odds Ratio (95%CI)** | **p-value (Prob>Chisq)** |
| --- | --- | --- |
|  |  |  |
| Male Gender  Age > 45.9  Location  intracranial vs. spinal      Primary vs. recurrent meningioma  NF2 vs. sporadic  CNS WHO grading  1 vs. 2  2 vs. 3  MIB1 >6.1%  CDK6 expression  p16 expression  pRB1 expression | 1.09 (0.84-1.41)  1.00 (0.74-1.36)  1.57 (1.06-2.34)  1.32 (0.89-1.96)  1.01 (0.48-2.15)  1.14 (0.83-1.55)  1.39 (0.43-4.51)  -  1.45 (0.90-2.34)  3.51 (2.8-4.39)  3.98 (2.85-5.57)  1.04 (0.82-1.33) | 0.526  0.974  0.026*  0.172  0.973  0.43  0.59  -  0.128  <0.001*  <0.001*  0.726 |

Table S5: Nominal logistic regression of factors associated with CDK6 expression.

|  | **Odds Ratio (95%CI)** | **p-value (Prob>Chisq)** |
| --- | --- | --- |
|  |  |  |
| Male Gender  Age > 45.9  Location  intracranial vs. spinal      Primary vs. recurrent meningioma  NF2 vs. sporadic  CNS WHO grading  1 vs. 2  2 vs. 3  MIB1 >6.1%  CDK4 expression  p16 expression  pRB1 > 1.4 | 0.78 (0.61-1.11)  0.83 (0.60-1.00)  1.99 (1.34-2.96)  0.64 (0.43-0.93)  1.78 (0.84–3.76)  1.15 (0.85-1.56)  0.48 (0.14-1.71)  0.54 (0.55–1.37)  3.51 (2.80-4.39)  1.13 (0.83-1.54)  1.24 (0.98-1.57) | 0.05*  0.214  0.001*  0.021*  0.13  0.365  0.259  -  0.868  <0.001*  0.438  0.072 |

Table S6: Multivariate Analysis of parameters associated with tumor recurrence including the p16/CDK-pathway

|  | **Hazard Ratio (95%CI)** | **p-value** |
| --- | --- | --- |
|  |  |  |
| Male Gender  Age > 45.59  Location  Convexity/Falx vs. spinal  Skull base vs. spinal    Simpson Grade ≤3  NF2 vs. sporadic  CNS WHO grading  1 vs. 2  1 vs. 3    MIB1 >6.1  Adjuvant Radiotherapy  CDK Pathway intact vs. discordant  CDK Pathway disrupted vs. discordant | 1.42 (1.11 – 1.83)  1.18 (0.88 – 1.57)  1.62 (0.82 – 3.21)  1.15 (0.58 – 2.29)  0.27 (0.22 – 0.35)  0.28 (0.09 – 0.89)  3.25 (2.46 – 4.29)  28.84 (15.06 - 55.24)  2.68 (1.89 – 3.82)  0.32 (0.20 - 0.51)  0.60 (0.38 - 0.96)  1.55 (1.21-1.98) | 0.005*  0.264  0.162  0.691  <0.001*  0.032*  <0.001*  <0.001*  <0.001*  <0.001*  0.032*  <0.001* |

Table S7: Univariate Analysis of parameters evaluating the risk of recurrence (log-rank test)

|  |  | **p-value** |
| --- | --- | --- |
|  |  |  |
| Male Gender  Age > 45.59  Location  NF2 vs. sporadic  Simpson Grade ≤3  CNS WHO grading  MIB1 >6.1  p16 expression  CDK4  CDK6  pRB1>1.4%  Adjuvant Radiotherapy |  | 0.001*  0.015*  0.055  0.007*  0.001*  <0.0001*  <0.001*  <0.001*  0.001*  0.410  <0.0001*  0.712 |
|  |  |  |
